# Non-Local Interaction via Diffusible Resource Prevents Coexistence of Cooperators and Cheaters in a Lattice Model

**DOI:** 10.1371/journal.pone.0063304

**Published:** 2013-05-17

**Authors:** David Bruce Borenstein, Yigal Meir, Joshua W. Shaevitz, Ned S. Wingreen

**Affiliations:** 1 Lewis-Sigler Institute for Integrative Genomics, Princeton University, Princeton, New Jersey, United States of America; 2 Department of Physics, Ben-Gurion University, Beer-Sheva, Israel; 3 Department of Physics, Princeton University, Princeton, New Jersey, United States of America; 4 Department of Molecular Biology, Princeton University, Princeton, New Jersey, United States of America; University of Maribor, Slovenia

## Abstract

Many cellular populations cooperate through the secretion of diffusible extracellular resources, such as digestive enzymes or virulence factors. Diffusion of these resources leads to long-range intercellular interactions, creating the possibility of cooperation but also the risk of exploitation by non-producing neighbors. In the past, considerable attention has been given to game-theoretic lattice models of intercellular cooperation. In these models, coexistence is commonly observed between cooperators (corresponding to resource producers) and cheaters (corresponding to nonproducers). However, these models consider only interactions between direct competitors. We find that when individuals are allowed to interact non-locally through the diffusion of a shared resource coexistence between cooperators and cheaters is lost. Instead, we find population dynamics similar to simple competition, either neutral or biased, with no balancing selection that would favor coexistence. Our results highlight the importance of an accurate treatment of diffusion of shared resources and argue against the generality of the conclusions of game-theoretic lattice models.

## Introduction

**Figure 1:**
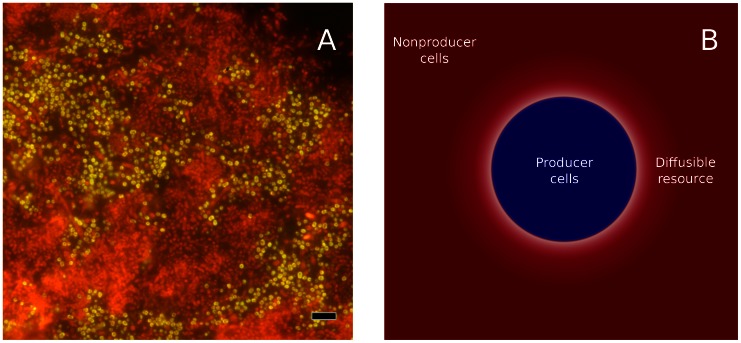
Diffusible resources. Microbes in biofilms often secrete extracellular resources despite the close proximity of unrelated cells. (a) A multi-species biofilm isolated from an extracted human tooth. *Streptococcus* sp. are shown in yellow, other species in orange and red; cells of *Streptococcus oralis* produce enzymes that release nutrients to all nearby cells [12]. Scale bar = 5 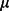
m. Figure from Vincent Zijnge [42]. (b) Cells (blue) release diffusible resources into the environment. These resources confer a growth benefit on all nearby cells, including non-kin nonproducer cells (red), potentially leading to the risk of exploitation of producers at domain boundaries.

A vast array of species employ diffusible extracellular factors to alter the local environment of their cells. Most multicellular organisms secrete digestive enzymes and acids in their digestive tracts. Both healthy and cancerous human cells secrete a host of signaling factors to regulate growth processes [1]. Microbes living in biofilms use diffusible molecules to degrade host tissues, digest nutrients, chelate metals, neutralize antibiotics, and sequester toxins [2]–[7] (Fig. 1a). In some cases, the processed substrate, rather than the extracellular factor itself is what diffuses [6]. In either case, diffusible resources help cells engineer their surroundings, providing the cells with a variety of benefits. However, in addition to conferring benefits on the producers, extracellular resources can confer a benefit on nearby, potentially unrelated cells (Fig. 1b).

Diffusible extracellular resources can find and interact with substrates that are inaccessible to the producing cell. For this reason, they have the potential to perform functions that private resources, even surface-bound extracellular factors, cannot. For example, the opportunistic human pathogen *Pseudomonas aeruginosa* exports the diffusible phenazine pyocyanin, which can act as a rudimentary circulatory system [8], as well as attack both host tissue and competing species of bacteria [9]. Notably, cells coordinate their pyocyanin production in response to that of other cells [10]. Other examples include the iron scavenging pigment pyoverdine [11] and enzymes such as exoglycosidases, which digest high molecular weight polysaccharides into simpler sugars [12].

Clearly, if the diffusion length is long and the cost of production is significant, nearby nonproducing cells can enjoy a competitive advantage over producers. Hence, an invader or a nonproducing mutant in a group of resource-producing cells may outcompete the producers, eventually leading to the loss of extracellular resource production in the population. How is it then that production of diffusible resources is widely observed, even among microorganisms in multispecies consortia [13]–[15]? In fact, the persistence of high genetic diversity in such consortia (e.g. dental biofilms) over long times suggests a mechanism for the coexistence of producers and nonproducers.

Highly detailed, ad-hoc individual-based models (IBMs) have been developed to study population dynamics in competitive cellular populations. For example, Xavier and colleagues developed an IBM for growth of multispecies biofilms featuring cell-cell adhesion and detachment, fluid transport, nutrient depletion and the transport of extracellular particles [16]. Recently, Momeni and colleagues explored a mutualistic interaction in yeast involving diffusible extracellular resources using both computational and experimental methods. Their IBM, which incorporated nutrient uptake, diffusion, and release, as well as cell division, death, and rearrangement, predicted that strongly interdependent mutualists would form alternating layers, consistent with their experimental results [17].

These individual-based modeling approaches facilitate a mechanistic understanding of the interaction between cells in specific microbial environments. For broader claims about the fate of cooperating populations, theorists have generally turned to spatial extensions of game-theoretic models. The two most broadly used classes of game-theoretic models are the Prisoner’s Dilemma and the Snowdrift Game. The Prisoner’s Dilemma (PD) is a pairwise interaction, or “game,” nominally involving two accused criminal confederates. In this game, the highest payoff goes to a defector whose opponent cooperates (does not defect); a cooperator whose opponent defects gets the lowest payoff. The second highest payoff is achieved when both players cooperate [18]. This is a good model for an interaction within which a resource is useful but not strictly necessary. By contrast, in the Snowdrift Game (SG), nominally based on a social impasse concerning who will shovel the snow, the worst payoff goes to mutual defectors (non-shovelers), and the second-worst payoff goes to a cooperator (shoveler) whose opponent defects [19]. This is a better model for situations in which a resource is essential for group survival, even though its production may be costly to the individual producer. For example, Gore and colleagues showed that SG is a good model for a well-mixed population of yeast cells producing an essential resource which is in part allowed to diffuse away from the producer [6].

These game-theoretic models have the advantage of having a well-defined optimal strategy for a single instance, greatly facilitating analysis over repeated encounters. However, their extension to space requires strong assumptions [20]. In non-spatial models, where any individual is equally likely to interact with any other individual, coexistence between pure-strategy cooperators and defectors is commonly seen in the SG case, or in cases of mutual dependence [6], [21], [22]. By contrast, stable coexistence of pure-strategy cooperators and defectors is impossible in a non-spatial PD interaction: defectors dominate cooperators, leading to the collapse of cooperation [18], [23]–[25].

Interestingly, these results are found to be nearly reversed in spatially structured models within which individuals compete only with their nearest neighbors. In these competitions, individuals are arrayed on a lattice, and play a pairwise PD or SG with each of their nearest neighbors; an individual’s fitness is a function of the sum of its payoffs from each pairwise interaction. A subset of cells are then replaced by fitter neighbors according to one of several update rules: one cell may be chosen at random to replace a neighbor (as in [26]), or each cell is replaced with a copy of the fittest individual in its neighborhood (as in [27]). The results are essentially equivalent. Namely, isolated PD cooperators are extremely unfit and are eliminated, while PD cooperators with at least some cooperator neighbors are able to survive. As a result, nearest-neighbor spatial PD models display coexistence through the formation of homogeneous cooperator groups. This coexistence can last indefinitely, even in the presence of stochastic fluctuations, and is often accompanied by the emergence of definite spatial structures. The outcome is quite different for nearest-neighbor SG competitions: the ability of individual SG cooperators to survive hampers their overall survival in nearest-neighbor spatial games. Unlike the original Nowak PD model [27], for which “winners” were chosen deterministically on the basis of greatest fitness, reproductive success in the Hauert SG model is chosen randomly, with the transition probabilities weighted by the payoffs. (Both pairwise interactions with a single neighbor and the sum of payoffs with all neighbors were used, with indistinguishable results.) Isolated cooperators are slightly less fit than the surrounding cheaters, but if they reproduce, the resulting cooperator pair is fitter than surrounding cheaters. These and other local effects lead to the tendency of cooperators to produce unstable dendritic structures that grow, collapse, and sometimes vanish locally. As a result, cooperators remain rare in the nearest-neighbor SG game, and can go entirely extinct for high cost-to-benefit ratios [26].

Nearest-neighbor interactions are easy to simulate and analyze. However, such models are unphysical in several ways with respect to microbial populations. First of all, such models evolve by winners replacing losers with copies of themselves. As such, no evolution takes place at the interior of a homogeneous group; rather, all competition must take place at the interface between populations. One consequence is that, in the short term, the dynamics in these models depend entirely on neighborhood composition at the competing fronts. On a rectangular lattice, a cell has only four neighbors, representing the totality of cells affecting its fitness. As a result, the transition of even a single neighboring cell can have a profound impact on the fitness of a cell relative to a neighboring competitor, while a cell two spaces distant is entirely irrelevant. While certain contact-dependent signaling processes do indeed have a strong effect on the behavior of nearest neighbors, most cooperative interactions involve alterations to the environment, which have a more diffuse neighborhood effect. Nowak and colleagues addressed this problem by weakening the dependence of fitness on local composition by randomizing the connectivity within the lattice in a PD model. They also examined interactions on a random lattice, in which cells were “neighbors” if they lay within a defined radius of one another. They found that coexistence was preserved, even when the effective group of “neighbors” extended to a larger area in this manner [28], [29]. These alternative topologies allow cells to compete with individuals who are not nearest neighbors. However, cell fate still depends entirely on fitness interactions with direct competitors. What happens when two individuals can affect one another’s fitness, even though they are not direct competitors?

To address this question, we developed a model in which cells interact within a diffuse neighborhood, but still compete for space with nearest neighbors as in previous studies. This model is intended to test the relevance of existing game-theoretic models to the problem of coexistence of producers and nonproducers in microbial communities. In our model, the producers (equivalent to the cooperators in a game-theoretic model) affect the fitness of nearby cells through the production of a diffusible resource. The resource confers a benefit both on the producing cell and on nearby individuals; additionally, there is a cost to the producer. Nonproducers enjoy the benefits of the resource without the production cost. Surprisingly, we find that this model exclusively exhibits the dynamics of simple neutral or biased competition. In particular, long-term coexistence of producers and nonproducers is never observed. This follows from the smoothing effect of diffusion on resource access, resulting in a homogenization of benefit. We conclude that non-local interactions among cells mediated by diffusible resources in a birth-death model can lead to qualitatively different dynamics than those observed in corresponding nearest-neighbor models. Our results highlight the importance of an accurate treatment of diffusion when modeling microbial populations.

## Model & Methods

The model is an adaptation of the fully occupied lattice models used in, e.g., [20], [26]–[28]. It consists of two coupled processes: the birth and death of individual cells, and the production and diffusion of a shared resource. To facilitate comparison with local competition models [20], [26]–[28], we model cells and resource concentrations on the same two-dimensional square lattice. To avoid edge effects, the lattice features periodic boundary conditions. Every lattice site is occupied by one of two cell types: "producer" (P) cells and "nonproducer" (NP) cells; no lattice site is permitted to be vacant. The cell types differ in their production of a diffusible resource. The resource is created only by P cells, but confers a benefit on both P and NP cells.

All cells have a basal growth rate 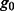, but P cells incur a reduction in growth rate, 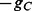, due to the cost of production. Hence the growth rates for P and NP cells, respectively, are given by:
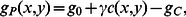
(1)

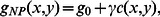
(2)where 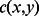 is the concentration of the diffusible resource at the discrete lattice site 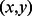. (Since resource concentration is represented on a lattice, 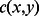 represents the mean resource concentration over the area of the cell at 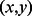.) Thus, the diffusible resource leads to a trade-off between the P cells, which have greater access to the resource, and the NP cells, which do not incur the production cost. Benefit from resource access is linearly proportion to resource concentration, with proportionality constant 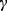. As shown in the Discussion, this assumption of linearity does not affect the behavior of the system.

Each producer generates a total flux 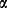 of the resource, which has diffusion constant 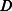, and is subject to first-order consumption/degradation at a rate 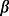. (Since all lattice sites are occupied, 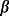 is a constant.) Hence the resource field resulting from a single P cell at 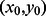 satisfies
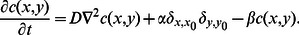
(3)Here 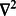 corresponds to the discrete 2D Laplacian:
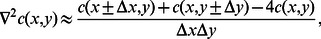
(4)where 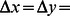 cell length.

**Figure 2:**
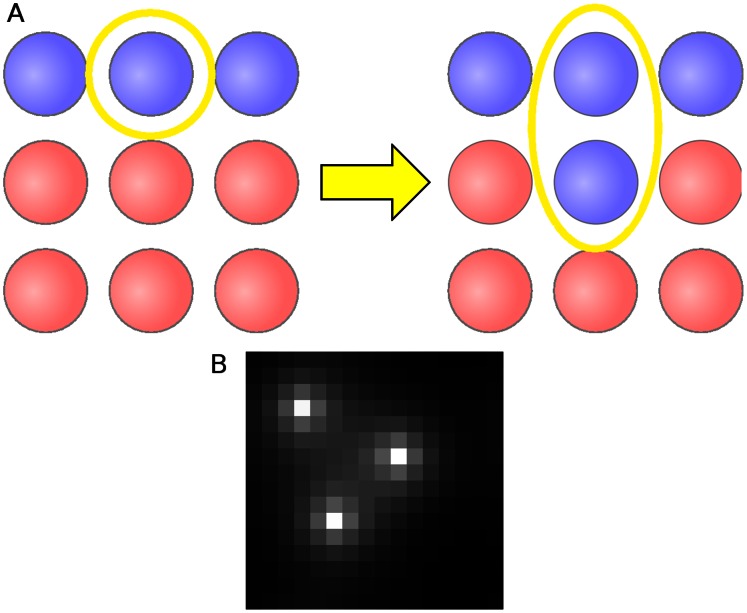
Model mechanics. (a) Stochastic birth-death model. Cells of two types - producers (blue) and nonproducers (red) - occupy all sites of a square lattice. Each time a cell divides, it replaces a randomly chosen neighbor with a daughter of its own type, leading to a stochastically evolving boundary between producers and nonproducers. (b) Steady-state distribution of diffusible resources around single producer cells, from Eq. 3 with 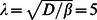.

In general, for biologically relevant regimes, the time scale of cell division greatly exceeds that of diffusion. Consider, for example, diffusion through the hydrogel matrix of a microbial biofilm. Diffusion constants for enzymes or other globular proteins through water are on the order 100 *µ*m^2^/sec; through the hydrogel matrix of a microbial biofilm they are on order 1 *µ*m^2^/sec [30]. One cell is on the order of 1 *µ*m. Hence, even for diffusion length 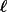 on the order 10 cell lengths, a diffusion process would reach steady state in seconds to minutes (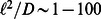 sec). By comparison, bacterial cell division takes tens of minutes to hours or longer, depending on conditions. Therefore, for each configuration of cells, we solve Eq. 3 in steady state, 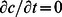. For a single P cell, we obtain a resource distribution that decays exponentially, with characteristic length 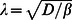. By linearity, the total resource concentration 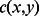 for a given configuration of producers is the superposition of such fields (Fig. 2b).

In the simulation, the configuration of cells changes stochastically as individual cells are chosen to divide and replace one of their four cardinal neighbors (Fig. 2a). Since replacement of a cell by another cell of the same type does not lead to a change in the configuration of cells, in practice, only “boundary” cells, which have at least one cardinal neighbor different from themselves, are allowed to divide. This is a spatial analog of the Moran process [31]. Algorithmically, we describe the process as follows:

1. Replace one cell with the daughter of a neighboring competitor. The probability of each possible replacement event is proportional to the growth rate of the dividing cell.
2. Calculate the stochastic waiting time since the previous event, based on the growth rate of all boundary cells in the system.
3. Recalculate the steady-state distribution of the diffusible resource, and the growth rates of all boundary cells via Eqs. (1–2).

The waiting time is determined via the exact Doob-Gillespie (DG) algorithm [32]. In a DG process, the waiting time between events 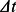 is Poisson distributed with a mean equal to the sum of all individual event rates:
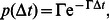
(5)where 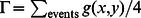 and 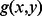 is the growth rate of the dividing cell in each replacement event in the sum. (Since each cell can initiate up to four events, the 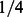 factor normalizes the per-event occurrence rate to the per-cell growth rate.)

### Parameters

The parameter space is simplified in several ways. First, we note that overall benefit from resource equals the concentration of resource 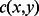 times the per-unit benefit 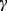. Since resource production rate 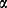 just sets the magnitude of 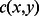, without loss of generality we can take 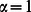 to be a basic unit of the system, leaving 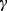 as a free parameter with which to adjust the resource benefit. Next, the model has two very different time scales - the time scale for division and the time scale for the equilibration of the resource. As discussed previously, cell division is far slower than resource diffusion (i.e. 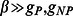). Hence we assume that the solute distribution instantly relaxes to a new steady state after each cell division. As such, we reduce consideration of the diffusion constant 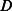 and decay rate 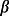 to a single dimensional combination: the diffusion length 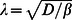. Without loss of generality, therefore, we may choose 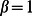.

We measure growth rate in units of the basal growth rate in the absence of any resource, i.e. 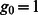. Next, we consider the cost of production 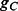. This parameter determines the upper bound on the fitness difference between producers and nonproducers. When 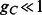, producers and nonproducers with similar resource access have essentially equivalent growth rates. Since resource diffusion smooths resource concentration at the competitive front, 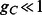 will lead to a trivial, nearly neutral dynamic, in which the two cell types are essentially equivalent and one will eventually fix due to enetic drift. To look for coexistence, we therefore consider the largest possible 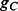, with the constraint that the growth rate for producers in the absence resource should not become negative. Hence we take 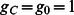.

### Neutral Model

As a negative control, in which long-term coexistence of cell types is impossible, we simulated a neutral birth-death model. In our framework, neutrality is achieved by setting the production rate 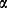 and the production cost 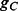 to zero, so there is no resource, and every replacement event has equal probability.

### Mutualism Model

As a positive control for coexistence, we consider two symmetric cell types each of which produces a diffusible resource that increases the growth rate of the *other* cell type. The growth rate of a cell at site 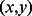 is therefore
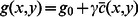
(6)where 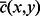 is the concentration of the resource produced by the complementary cell type. In this model, the fastest growing cells are those nearest to the complementary cell type, leading to selective pressure toward a balanced, intermixed population. This control is a two-dimensional analog to the mutualism model of [33].

### Radial Distribution Function

To characterize spatial structure, we evaluated the radial distribution function. Specifically, for a given configuration of cells, the distribution of cell types was considered as a function of distance from each NP cell. The RDF is the fraction of cells that are NP, as a function of Euclidean distance from the central NP cell, averaged over all NP cells. Typically, the RDF was averaged over multiple configurations, with each configuration weighted by its number of NP cells. For the neutral and mutualism models, since both types of cells are equivalent, the central cell type for the RDF was chosen arbitrarily.

## Results

In nearest-neighbor spatial games, such as [26], [27], individuals can only affect the fitness of direct competitors. For a broad range of conditions, these nearest-neighbor games give rise to coexistence between cooperators and cheaters, including definite spatial structures of cooperators and cheaters. However, in cellular populations such as bacterial biofilms and multicellular tissues, individual cells engineer their environment by exporting diffusible extracellular factors. These factors affect the fitness of not just a producer’s direct competitors, but of many cells in its vicinity. When individuals are allowed to interact at long range with non-competitors is coexistence preserved? More specifically, what is the effect of diffusible resources on the spatial structure of the population?

In order for long-term coexistence of producers (P) and nonproducers (NP) to occur in a competition model, a conditional selection bias must act whenever the distribution of cells deviates from a coexistent steady state. This sort of selection bias, called “balancing selection,” necessarily leads to a growth bias in favor of the underrepresented cell type. That is, when P cells become too frequent (i.e., the fraction of P cells exceeds the preferred fraction), NP growth would be favored; conversely, when P cells become too infrequent, P cells would be favored. Furthermore, this balancing selection must be strong enough to overcome the tendency of random fluctuations to drive the system toward the fixation of one cell type. In our lattice model, only cell divisions at the boundary between producers and nonproducers affect the distribution of cells (Fig. 2a), so any balancing selection must act at the boundaries.

**Figure 3:**
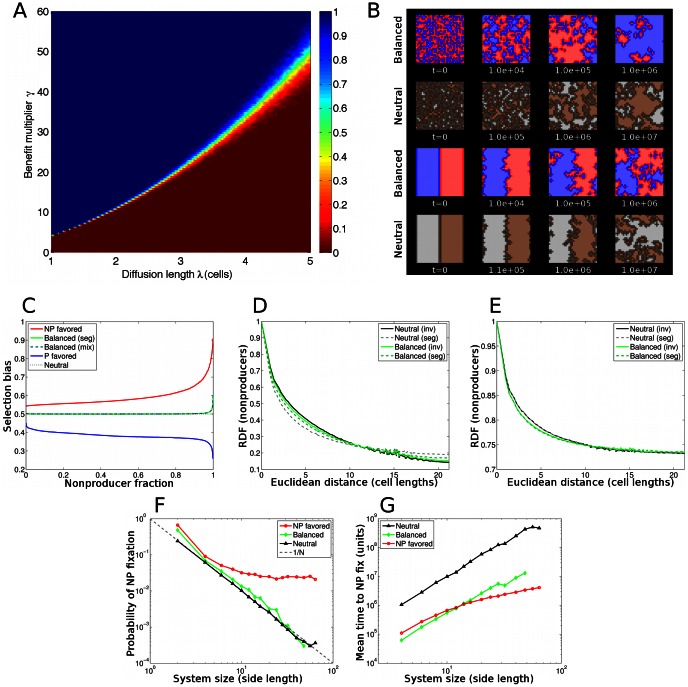
Competition model results. (a) Dependence of probability of cooperator fixation on diffusion length 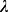 and benefit value 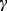. Interpolated from 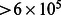 simulations. Each simulation was initialized with equal numbers of randomly arranged P and NP cells in a 32×32 system and run until one cell type reached fixation. (b) Time series of simulations. Cell types begin at equal frequency, with either well-mixed (upper) or fully segregated (lower) initial conditions. First and third rows show a diffusible resource model with producers (blue) and nonproducers (red). Parameters: diffusion length 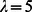 and benefit multiplier 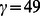 for balanced competition. Second and fourth rows show a neutral model. Boundary cells are shown in dark colors. The diffusible-resource model and the neutral model exhibit similar dynamics. (c) Selection bias, i.e. probability that next event will be a nonproducer replacing a producer, as a function of nonproducer fraction. In the neutral case (dashed black curve), selection bias is exactly 0.5 by construction. NP- and P-favored simulations began with a single invader cell of the favored type. Balanced results began with equal populations in either the segregated (solid green) or well-mixed (dashed green) initial conditions shown in (b); the results are indistinguishable. Parameters: Blue curve: 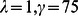. Red curve: 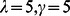. Green curves: 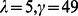. Non-neutral curves are averaged over at least 500 simulations apiece. Radial distribution function of nonproducers at (d) 25% and (e) 75% nonproducer fraction. Parameters as in (b). Initial conditions are a single nonproducer invasion (solid curves) or two segregated equal populations (solid curves). Also shown is the radial distribution function for the neutral model for both single-invader and segregated initial conditions. Results are averaged over at least 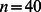 observations in each case. Probability of nonproducer fixation, starting from a single invading cell, as a function of linear system size. The neutral model (black) exhibits the expected fixation probability 1/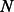 for 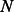 equivalent cells. Diffusible-resouce model with balanced parameters also exhibits 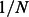 scaling (green curve: 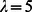, 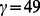, as in (b)). For NP-favored case, fixation probability saturates as expected for a faster growing invader (red curve: 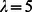, 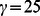). Results are averaged over at least 15,000 simulations for neutral and balanced models, and 2,000 simulations for NP-favored. (g) Average time to fixation of a nonproducing invader (for cases in which nonproducers fix), as a function of linear system size.

In looking for this balancing selection, we found that, for a given resource diffusion length 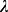, there is a transition from P to NP dominance around a critical benefit value 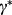. This fitness transition does not depend on initial conditions. Fig. 3a shows the effect of diffusion length 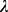 and benefit value 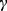 on the probability of producer fixation, given a 50–50 initial distribution of cooperators and cheaters. When the diffusion length is very short (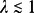 cell length), the resource is essentially private, and producers are effectively not cooperating. This leads to a simple fitness difference that depends only the cost-benefit ratio of resource production. On the other hand, when the diffusion length is long (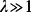 cell length), resource distribution is nearly uniform. Since nonproducers have only slightly less resource access than producers, a change in 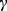 has a relatively small effect on individual fitness at the front. As a result, the transition from P- to NP-domination is gradual at long diffusion lengths, with intermediate values having the potential for either outcome. Therefore, for a given diffusion length 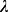, we found the critical value of 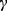 at which the two fixation states were equally probable, and identified this as the most likely 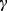 value for balancing selection. An analysis of this critical value of 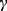 versus 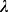 is given in the SI.).

Figure 3b shows the time evolution of a balanced P-NP competition model, with 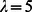 and 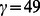, and a neutral model, starting from either a well-mixed or a fully segregated initial condition. In all cases, the initial frequencies of the two cell types are equal. For the P-NP model, domains of like cells form through random fluctuations at the boundaries. Smaller domains are subsumed by larger ones until eventually the entire system becomes a single domain of one cell type. This long-term behavior is independent of initial conditions. Moreover, there is a strong resemblance in dynamics between the balanced P-NP model and the neutral model. In fact, the only distinguishing feature between these two models is the time scale: in the absence of the diffusible resource, the neutral system evolves roughly ten times more slowly. This difference in time scales is due to the benefit conferred by the diffusible resource: with the balanced benefit value 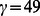, one unit of the resource speeds growth by a factor of 49 over the basal rate 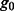. For zero diffusion, and with 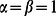, the resource concentration is 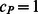 for P cells and 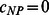 for NP cells; for infinite diffusion, the concentration 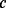 scales with P fraction. Hence, for the relatively long diffusion length 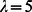, we observe a concentration of approximately 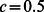 for an initial 50–50 distribution. Hence, given the balanced parameters 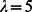, 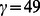 described earlier, the absolute growth rate for all boundary cells in the P-NP model is around 25 times higher than for a neutral model.

The neutral-like population dynamics of the balanced P-NP model are illustrated in Fig. 3c, which shows selection bias as a function of NP frequency. The selection bias is defined as the mean probability, given a specified NP population fraction, that an NP cell will replace a P cell in the next replacement event. Note that for balanced parameters the selection bias is 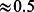, *i.e.*, producers and nonproducers are equally favored, regardless of NP fraction, until just before NP reaches fixation. This is true whether the cells begin segregated or well-mixed. By contrast, coexistence requires a selection bias that acts to restore the system to a preferred state.

Balancing selection is absent even for very short diffusion lengths, where one might have anticipated the existence of spatial configurations that strongly favor each of the two cell types (Fig. S3). Strong producer bias is only possible when the diffusion length is short and the benefit of the resource is high compared to the cost; otherwise, the behavior is essentially neutral or biased toward nonproducers.

Does the absence of global selection bias in the balanced case imply neutral-like spatial dynamics as well? It is conceivable that the spatial configuration of cell neighborhoods could give rise to locally frequency dependent non-neutral dynamics, even if the population shows no overall selection bias. If this were the case, one would expect the spatial structure of the balanced P-NP and neutral models to differ. To investigate this possibility, we evaluated the radial distribution function (RDF) of both the balanced and neutral cases at the same producer fraction. The remarkable similarity of the RDFs, shown in Figs. 3c,d, strongly suggests that there is no significant difference in emergent spatial structure between the two models. Namely, the RDF exhibits the same approximately exponential decay with distance (Fig. S4) for both models. Thus, the quantified spatial behavior exhibited by the P-NP model in the balanced regime can be fully attributed to neutral competition.

Next, we asked whether the initial condition had any effect on the long-term spatial configuration of the system. To find out, we measured the RDF in cases where a single NP invader had grown to either 25% fraction or 75% fraction. These are relatively rare events. By comparison, a 50–50 initial condition will always reach either a 25% or 75% NP fraction eventually. Nevertheless, the RDF curves for the rare cares where a single NP invader achieves 25% or 75% fraction are very similar to the corresponding curves for a 50–50 initial distribution, indicating that initial conditions have no effect on the long-term spatial configuration. Indeed, by the time the single NP invader has taken over 75% of the system, the RDFs for the single-invader and 50–50 initial conditions are almost identical.

Could the similarity between the balanced and neutral spatial dynamics be an artifact of small system size? To ascertain whether this was the case, we characterized the long-term dynamics of the balanced and neutral models, as well as of a weakly NP-biased model, over a range of system sizes. Fig. 3f shows the probability of NP fixation, starting from a single NP invader, as a function of linear system size. We find that these dynamics are almost entirely consistent with an equivalent well-mixed model, suggesting that spatial configuration plays a negligible role in the dynamics. This is consistent with our expectation that resource diffusion homogenizes resource distribution along the competitive front.

Is the time to fixation in the balanced P-NP model also consistent with neutral-like dynamics? In the neutral case, time to fixation scales with linear system size (Fig. 3g). As discussed previously, the diffusible resource increases the overall growth rate of cells; however, with balanced parameters, the relative growth rates of producers and nonproducers remain equal (Fig. 3d). Hence, one expects time to fixation to exhibit the same scaling as the neutral process, albeit with a smaller prefactor. This is precisely what we see in Fig. 3g. Moreover, a weak NP advantage leads to accelerated fixation time at large system sizes, consistent with our expectation that invader growth is steady and deterministic once the establishment population of a faster-growing invader is reached [34]. The results for the fixation time further demonstrate that, far from enabling coexistence, diffusible resources drive the system toward the fixation of one cell type.

**Figure 4:**
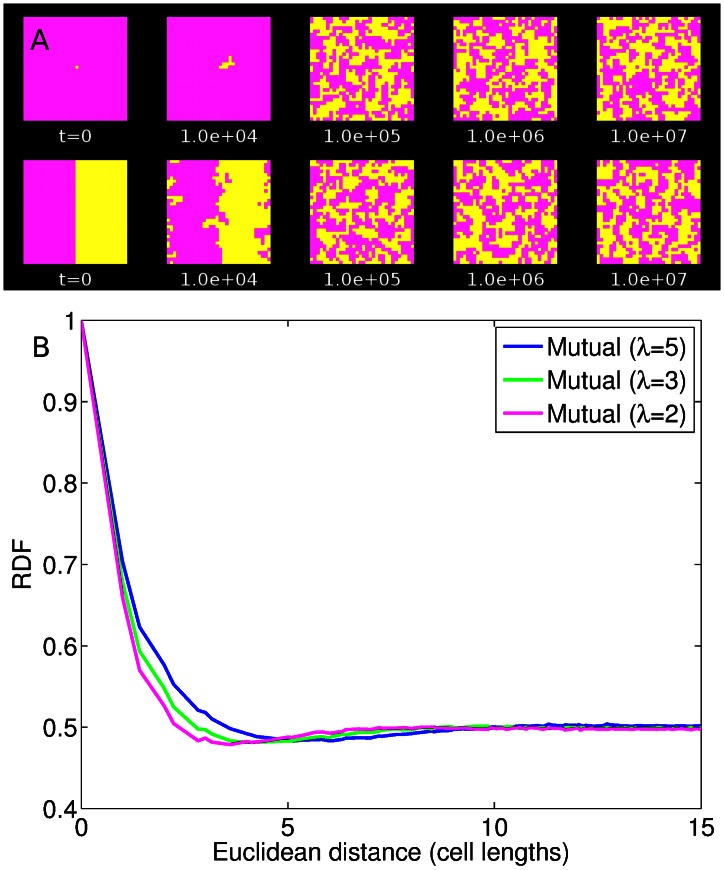
Mutualism model results. (a) Time series of mutualism model with diffusible resources. Initial conditions: single-cell invasion (upper) and segregated, equal domains (lower). Parameters (both cell types): 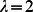, 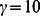. (b) Radial distribution function for the mutualism model at steady state. Diffusion length 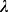 as shown; other parameters as in (a). Cell type is correlated and then uncorrelated for a longer interval, reflecting the spatial structure of kin domains. Neutral RDF calculated at a 75%/25% distribution starting from a single-invader initial condition. Results are averaged over 200 simulations for each diffusion length 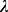.

The conspicuous absence of stable, finite-scale spatial structures in the diffusible-resource model is striking. In previous local-interaction models, producers and nonproducers have been observed to self-organize into finite groups of like individuals that persist for long times. Could we have somehow failed to recognize stable coexistence in our model? To rule out this possibility, we sought to establish that local competition with diffusible resources can, under the right circumstances, lead to readily recognizable coexistence within our modeling framework. To this end, we developed a “positive control” for coexistence - a mutualistic interaction with diffusible resources. In this mutualism model, two cell types each produce a diffusible resource that confers a benefit only on the other cell type. Cells at the boundary still compete, but now their growth rate depends on the frequency of the other cell type within the diffusion length of the resource. The two cell types shown in Fig. 4 are equivalent but complementary: the cost of production 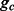 (set equal to basal growth rate 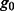) and resource benefit 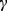 are identical. Strikingly, both coexistence and finite-scale spatial structure are readily observed in the mutualism model. In the example in Fig. 4a, the diffusion length is 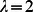 and the benefit to complementary cells is 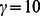. The result is a definite spatial structure consisting of finite-sized kin domains, independent of initial conditions. This strong spatial patterning is evident in the RDF (Fig. 4b), where cell type is strongly correlated at short lengths and then uncorrelated at long lengths.

## Discussion

We investigated the possibility that interaction via diffusible resources can lead to long-term coexistence between producers and nonproducers in a lattice model. We explored this hypothesis using a model in which producers (P) and nonproducers (NP) divide and replace one another, with a growth rate determined by access to a resource produced only by P individuals. We compared these results with a neutral model, in which cells are chosen to divide and replace one another entirely at random, and with a mutualism model, in which each cell type produces a diffusible resource used only by the other cell type. We found that in the P-NP model the population structure evolves by either a neutral or biased drift process. For given parameters, either one cell type is favored or the competition is neutral for all initial conditions and population fractions. Balancing selection, which would restore the system to a favored mixed state, i.e., a state of coexistence, is never observed. Instead, cell groups fluctuate randomly in size until one cell type takes over the system, in a manner essentially identical to the behavior of a neutral or biased drift model. This outcome is striking, because it lacks the persistent spatial structures observed in previous “spatial game” models [20], [26]–[29].

What causes this drift-like dynamic in our P-NP competition model? The growth rate difference between P and NP cells is greatest at the interiors of their respective kin domains. However, all competition takes place at the boundaries. When diffusion lengths are long compared to a cell length, spatial variations in resource concentration are always gradual, meaning that the difference in resource concentration on the P and NP sides of a boundary is necessarily modest. Moreover, diffusion smooths out variations in resource concentration due to boundary shape. Critically, therefore, P and NP cells at the boundary receive nearly identical benefits due to resource concentration. Since all competition takes place among these boundary cells, spatial structure plays no meaningful role in the dynamics of the system for diffusion lengths long compared to a cell length.

By contrast, when diffusion lengths are short, the difference in resource access between P and NP cells can be substantial. In the latter case, however, interior P cells are unable to provide resources to their kin at the boundary. Instead, individual producers keep most of the resource for themselves (in effect, resources are no longer diffusible). Consequently, the competition becomes essentially frequency-independent; producers have a systematic advantage if and only if the resource benefit exceeds the cost. In either regime of diffusion lengths, no long-term coexistence is possible.

Consequently, the system evolves according to one of two relatively simple dynamics: neutral drift or simple biased competition. Simple biased competition can occur when either the resource is very costly or diffusion lengths are short; in all other cases, a neutral drift dynamic occurs. When the diffusion length is long, producers can at best compete neutrally. If the benefit is low, producers grow more slowly because of the cost of production; if the benefit is high, producers gain little advantage because nonproducers have almost as much access to the resource as producers. When the diffusion length is short, the two strategies represent a direct trade-off: P cells have greater resource access, while NP cells have faster basal growth. In this case, the cost-benefit difference determines a simple growth advantage for one cell type or the other, while zero cost-benefit difference results in neutral drift.

Could a saturating resource uptake curve, such as the Michaelis-Menten model used in [6], [17], facilitate coexistence? In fact, the answer is no. Consider the following nonlinear variants of the equations for diffusion (Eq. 3) and growth (Eqs. 1 and 2):
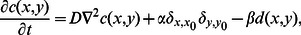
(7)

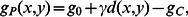
(8)

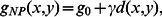
(9)where



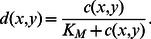
(10)In the limit 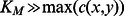, the equations revert to their originals. At lower values of 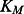, for which uptake saturates, cells near producers retain less of the resource. As a result, the solute concentration gradient around each producer is even shallower, resulting in an even more homogeneous solute distribution. Ultimately, therefore, saturating resource uptake has the effect of exacerbating the degeneracy of the dynamics.

Our results underscore a general shortcoming of neighborhood competition models, including spatial game models [20], [26]–[29]: the interior of a cell group plays no dynamical role whatsoever. By contrast, microbial systems *in vivo* often exhibit dynamics that are driven by internal cells. For example, Xavier and Foster found that extracellular matrix production by internal cells in biofilms drives outer cells towards the resource-rich frontier, enhancing growth rates [35]. More recently, Koschwanez and colleagues found that, among yeast cells producing an extracellular shared good, multicellular aggregates could survive at low-nutrient conditions whereas free-living cells could not [36]. Finally, slow-growing interior cells can drive the recovery of a microbial population following antibiotic stress [37]. Consequently, the dynamics of neighborhood competition models have little relevance to typical microbial populations.

In fact, coexistence of social strategies may not depend on direct interaction of “cooperators” and “cheaters” in general. Direct interaction occurs only when individuals are in close proximity; i.e., they compete directly. In a population of cells (e.g., a biofilm), this occurs only where the population has reached such a density that cells neighbor individuals of competing lineages. Within such a densely populated area, cells are limited for nutrients and space, with the result being that population dynamics are slow compared with the dynamics of the growing frontier. As a consequence, in many cases dynamics at the growing front are likely to drive the evolution of the population overall.

Even if nonproducers have a local advantage in a saturated population, producers may have an advantage during periods of population expansion. Indeed, Chuang and colleagues showed that bulk growth can favor producers even while local competition favors nonproducers [38]. Importantly, as shown, e.g., by Korolev and Nelson [39], mixed populations tend to segregate during periods of spatial expansion. This segregation creates distance between different growing lineages, reducing nonproducer access to diffusible resources. As a consequence, the benefits of diffusible resources can feed back primarily to producers, resulting in a much greater fitness differential between the strategies that ultimately favors producers.

It has also been suggested that intercellular signaling systems, such as quorum sensing in bacteria, may facilitate the evolution of cooperative behaviors [40]. What impact would quorum-sensing regulation have on the population dynamics of our local competition model? Our data suggest that quorum sensing is unlikely to suffice on its own to favor cooperation in a saturated habitat. For a saturated population, diffusible resources are least useful at the interior of a producer group, where population density is highest but growth is slowest. Meanwhile, at the edge of the producer group, resource production also confers a fitness advantage on nonproducers. In other words, neither positive nor negative regulation of resource production in response to cell density will facilitate the survival of cooperators in saturated populations. Additionally, quorum sensing introduces an additional vulnerability to exploitation by cells that produce the signal, but not the diffusible resource [40], [41]. Instead, we predict that the main advantages to producers of diffusible resources must accrue during periods of overall population growth. In this regime, quorum sensing can enhance producer fitness by reducing wasteful production.

## Conclusion

Competition between cooperators and cheaters at a cellular level has been modeled via spatial analogues of game-theoretic evolutionary systems. However, many well-known lattice models are based on such games allow only nearest-neighbor interactions. This restriction is unphysical in several ways. Most importantly, cells at the interior of a homogeneous population are excluded from contributing to the population dynamics, and the significance of local geometry near the boundary between "cheaters" and "cooperators" is exaggerated When these assumptions are relaxed to allow non-local interactions via diffusion of resources, the most notable features of these spatial games are lost. In particular, no coexistence is possible under any parameter regime. As a consequence, such spatial games may have less relevance for physical populations than previously assumed.

## Supporting Information

Figure S1
**Resource distribution.** Resource distribution around a point source for 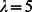. Red curve: resource distribution for the rectangular lattice used in the simulation. Dashed black curve: analytical result from Eq. S6. As in the main paper, 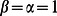.(TIFF)

Figure S2
**Effect of initial condition on long-term dynamics.** Time series of producer-nonproducer competition model with balanced parameters and short diffusion length 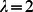, starting from multiple initial conditions. Producers shown in blue, nonproducers in red; boundary cells shown in dark shades. Parameters: 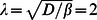, 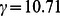.(TIFF)

Figure S3 **Single producer invasion of nonproducers.** Time series of producer-nonproducer competition model starting from a single producer cell. Parameters as in Fig. S2.
(TIFF)

Figure S4
**Selection bias versus nonproducer fraction.** Selection bias as a function of NP fraction at multiple values of 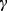 and for three initial conditions. Hue indicates overall bias. Bright red: 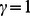. Bright blue: 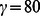. Solid lines, segregated equal domain initial condition. Dashed lines, single P invader. Dot-dash lines, single NP invader initial condition. Results averaged over 500 simulations for each curve.(TIFF)

Figure S5 **Spatial correlation in neutral model.** Radial distribution function (RDF) for neutral model, with reference cell type frequency at 75% (single invader curve from Fig. 3e). Fit line is an exponential function.
(TIFF)

File S1 **Analytical predictions.**
(PDF)
