# Supplementary figures and images for: Non-Local Interaction via Diffusible Resource Prevents Coexistence of Cooperators and Cheaters in a Lattice Model
Source: PLoS One. 2013 May 17;8(5):e63304. doi: 10.1371/journal.pone.0063304 (PMC3656920; doi:10.1371/journal.pone.0063304)

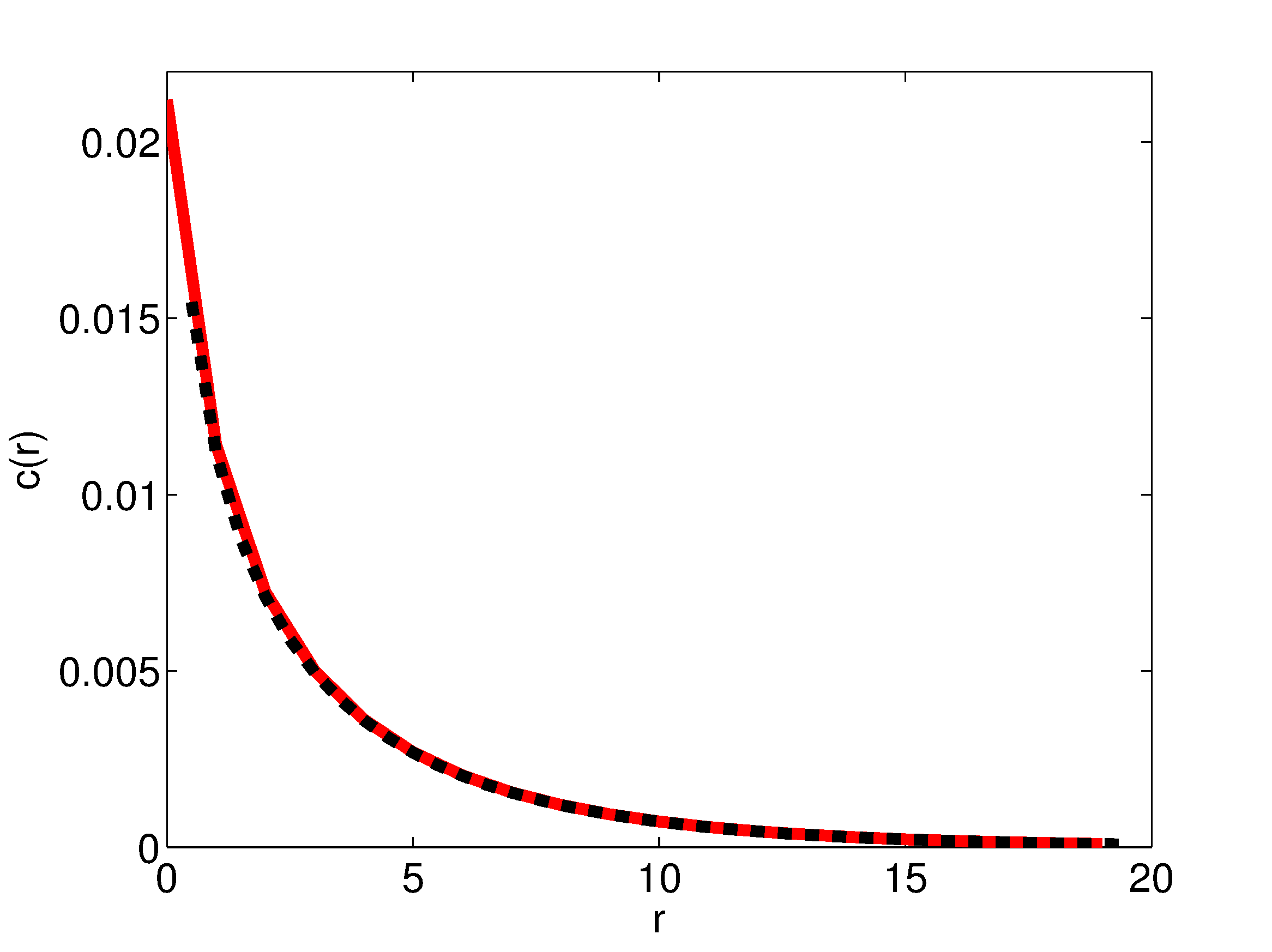

Supplement: Figure S1 — Resource distribution. Resource distribution around a point source for . Red curve: resource distribution for the rectangular lattice used in the simulation. Dashed black curve: analytical result from Eq. S6. As in the main paper, . (TIFF) [file pone.0063304.s001.tiff]

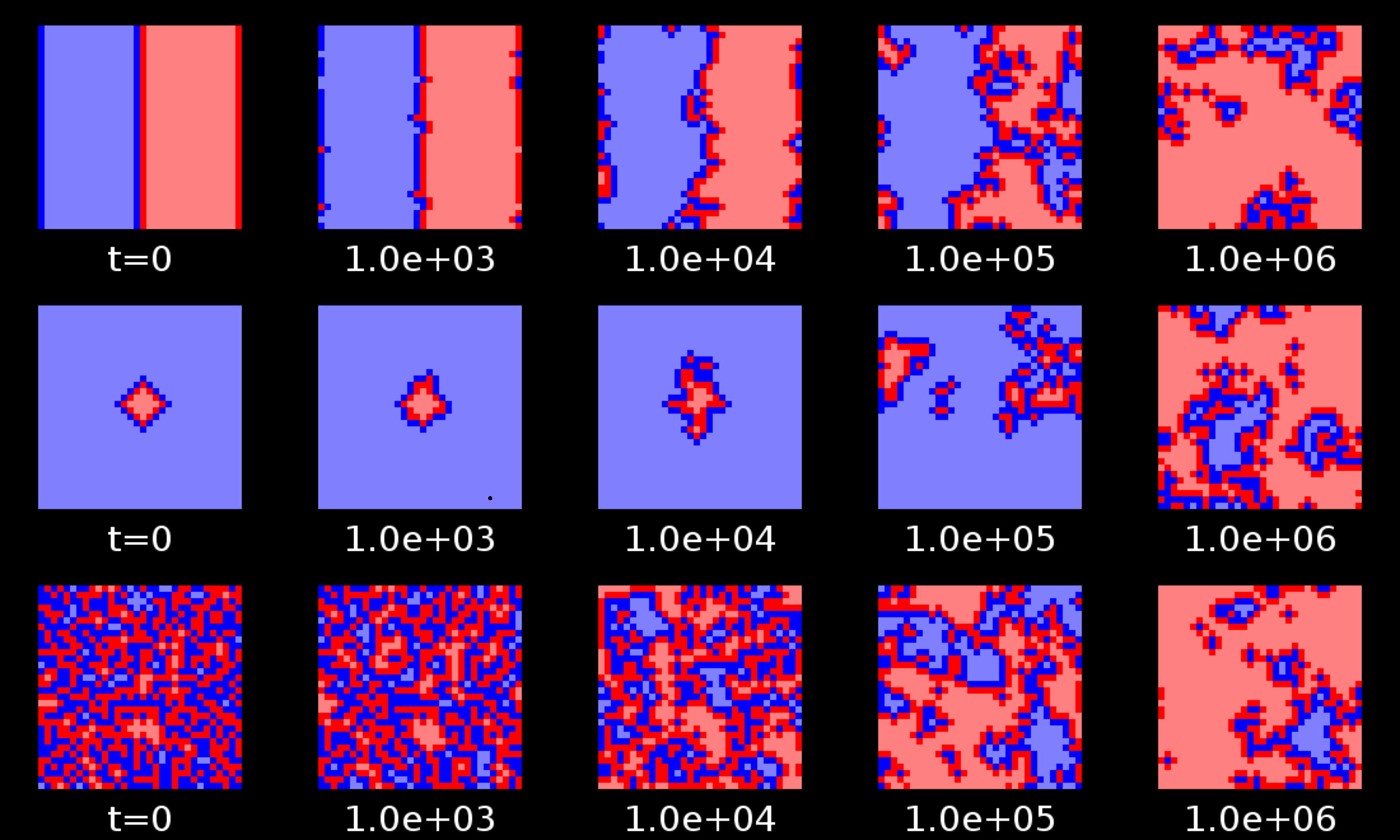

Supplement: Figure S2 — Effect of initial condition on long-term dynamics. Time series of producer-nonproducer competition model with balanced parameters and short diffusion length , starting from multiple initial conditions. Producers shown in blue, nonproducers in red; boundary cells shown in dark shades. Parameters: , . (TIFF) [file pone.0063304.s002.tiff]

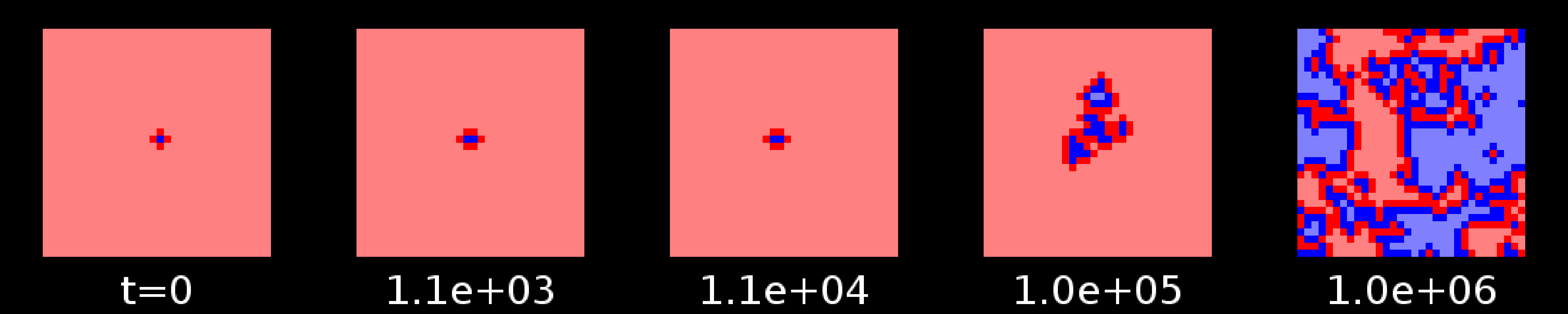

Supplement: Figure S3 — Single producer invasion of nonproducers. Time series of producer-nonproducer competition model starting from a single producer cell. Parameters as in Fig. S2. (TIFF) [file pone.0063304.s003.tiff]

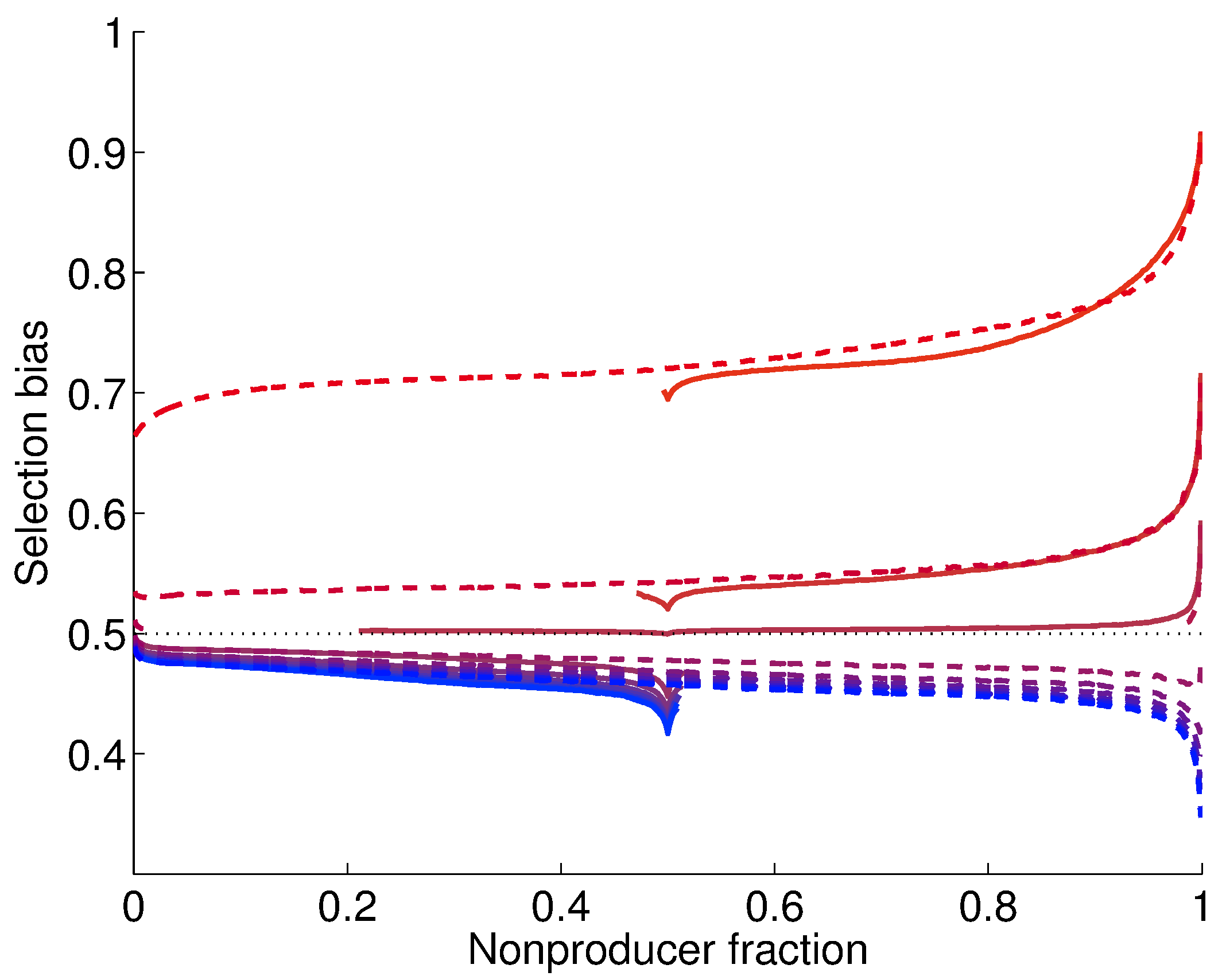

Supplement: Figure S4 — Selection bias versus nonproducer fraction. Selection bias as a function of NP fraction at multiple values of and for three initial conditions. Hue indicates overall bias. Bright red: . Bright blue: . Solid lines, segregated equal domain initial condition. Dashed lines, single P invader. Dot-dash lines, single NP invader initial condition. Results averaged over 500 simulations for each curve. (TIFF) [file pone.0063304.s004.tiff]

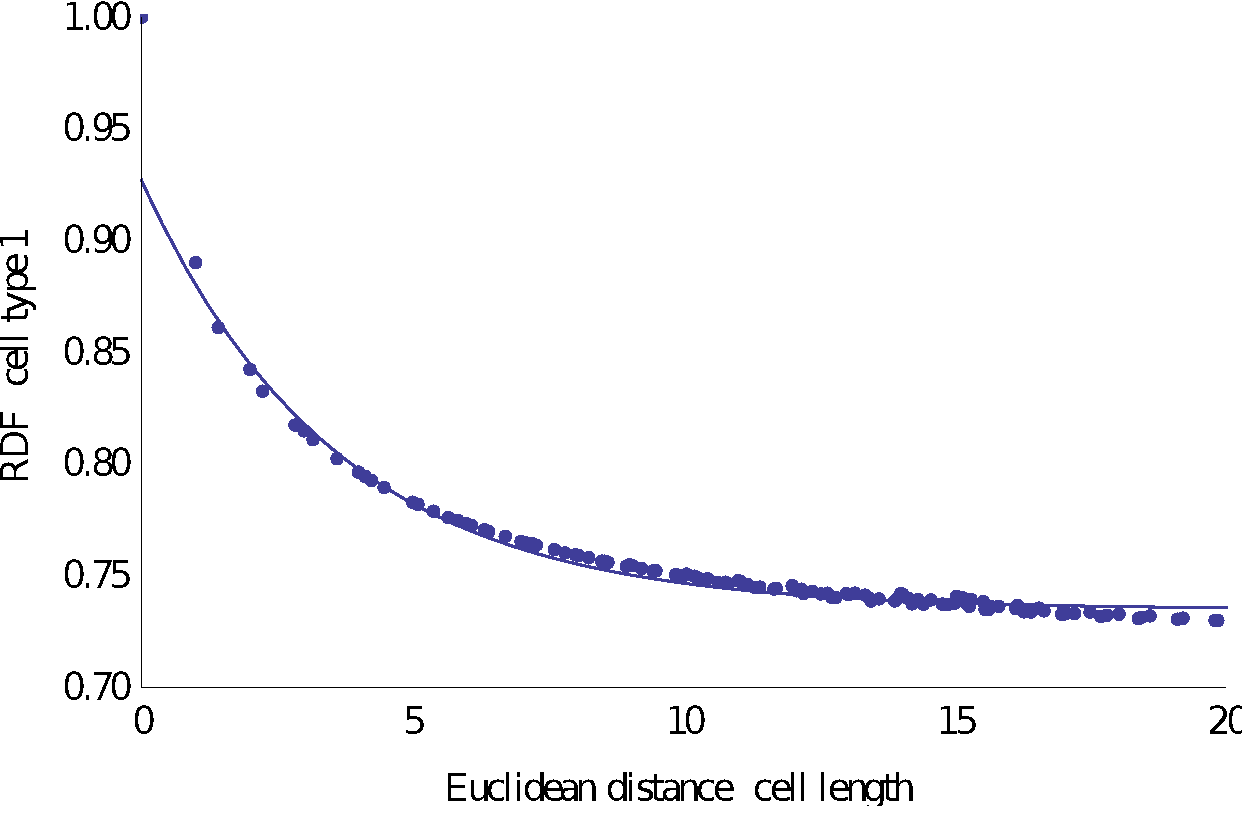

Supplement: Figure S5 — Spatial correlation in neutral model. Radial distribution function (RDF) for neutral model, with reference cell type frequency at 75% (single invader curve from Fig. 3e). Fit line is an exponential function. (TIFF) [file pone.0063304.s005.tiff]
