# Supplementary material for: Non-Local Interaction via Diffusible Resource Prevents Coexistence of Cooperators and Cheaters in a Lattice Model
Source: PLoS One. 2013 May 17;8(5):e63304. doi: 10.1371/journal.pone.0063304 (PMC3656920; doi:10.1371/journal.pone.0063304)
Supplement: File S1 — Analytical predictions. (PDF) [file pone.0063304.s006.pdf]

Supporting information for 'Non-local interaction via diffusible resource prevents coexistence of cooperators and cheaters in a lattice model'

David Bruce Borenstein[1], Yigal Meir[2], Joshua W. Shaevitz[1,3], Ned S. Wingreen[1,4]
1 Lewis-Sigler Institute for Integrative Genomics, Princeton University, Princeton, NJ, USA.
2 Department of Physics, Ben-Gurion University, Beer-Sheva, Israel
3 Department of Physics, Princeton University, Princeton, NJ, USA
4 Department of Molecular Biology, Princeton University, Princeton, NJ, USA
∗ E-mail: wingreen@princeton.edu

Relationship of dynamics to non-spatial model

For the neutral case of our P-NP lattice model, the probability of single-invader fixation is simply $1/N$, where N is the total number of cells, as expected for unbiased evolution. Notably, the same $1/N$ scaling is observed at all system sizes for the P-NP diffusible-resource model with balanced parameters, indicating that the dynamics are essentially neutral independent of system size. To confirm that our system size is large enough to capture non-neutral behavior, we chose parameters to slightly favor the NP invader. Because the bias is weak, the NP invader is still likely to go extinct due to stochastic fluctuations when its population is small compared to the overall population. However, once the invading NP cells reach a critical number (the "establishment" population), they are more likely to keep increasing in number than to fluctuate away. The result is a crossover from $1/N$ to N-independent fixation probabilities. This is analogous to a dynamic observed previously in a non-spatial, stochastic ODE model of mutation and selection. Namely, alleles with a weak fitness advantage Δs have a $1/N$ fixation probability for small N; however, once they have reached a critical number $\sim 1/\Delta s$, their population grows in a roughly deterministic way, because fluctuations are small compared to the overall trend of exponential increase [?]. We clearly see the transition to deterministic, system-size independent growth for the NP-favored curve in Fig. 3f, suggesting that even a weak frequency-dependent selective bias would be apparent in our balanced-P-NP-model simulations.

Analysis of invasion conditions

In order to understand the transition from P- to NP-dominated parameter regimes, we consider the conditions required for a single P cell to be more likely to divide than to be replaced by a neighboring NP cell.

If the producer is chosen to divide, it is certain to replace a neighboring NP cell. If any of its four cardinal neighbor nonproducers are chosen, the chosen cell will have a one in four chance of replacing the producer. Since the probability of selecting a cell for division is proportional to its growth rate (Eqs. 1 and 2), we have the inequality

$$(1)(1) \cdot [\gamma c(0,0) + g_0 - g_C] > (4)(0.25) \cdot [\gamma c(1,0) + g_0] \,. \tag{S1}$$

Simplifying this expression, we obtain

$$c(0,0) - c(1,0) > \frac{g_C}{\gamma}. \tag{S2}$$

Thus the critical value of γ^*, at which the cooperator is more likely to replace a neighbor than to be replaced, is

$$\gamma^* = \frac{g_C}{c(0,0) - c(1,0)}, \tag{S3}$$

The solute diffuses radially from the center of the producing cell and is subject to decay. Expressing this process in polar coordinates, at steady state we have

$$Dr\frac{dc(r)}{dr} + Dr\frac{d^2c(r)}{dr^2} - \beta r^2 c(r) = 0, \tag{S4}$$

$$\lim_{r\to\infty} c(r) = 0,$$
$$\lim_{r\to 0} J(r) = \alpha$$

where $c(r)$ is the concentration of resource as a function of distance from the source (i.e., the producer), and $J(r)$ is the total outward flux at radius r. This is a modified Bessel's equation with $p = 0$ and no increasing component. Therefore the solution will be a modified Bessel function of the second kind, $K_0(r)$. We use the limit of small radius r to find the particular solution

$$c(r) = \frac{\alpha}{2\pi\beta\lambda^2} K_0(r). \tag{S5}$$

We find that this result has excellent agreement with our lattice model, as shown in Fig. S1. Due to rotational symmetry, we can approximate $c(0,0)$ and $c(0,1)$ as the mean of a disc of radius $r = 0.5$ centered at the origin and of an annulus from $r = 0.5$ to $r = 1.5$, respectively. Substituting these integrals into Eq. S3, we find that

$$\gamma^* \propto \frac{\beta\lambda^2 g_C}{\alpha}. \tag{S6}$$

This result is consistent with the phase transition observed in Fig. 3a, in which the critical benefit value γ^* grows as the square of the diffusion length λ.

References

1. Desai MM, Fisher DS (2007) Beneficial mutation selection balance and the effect of linkage on positive selection. Genetics 176: 1759–98.
